# Chitosan-Branched Polyethyleneimine Hybrid Cationic Layer as an Effective Solution Towards Fire Vulnerability of Cotton Fabric

**DOI:** 10.3390/polym17243313

**Published:** 2025-12-15

**Authors:** Hamid Hassan, Zeeshan Ur Rehman, Jin Doo Yoon, Seung Woo Park, Bon Heun Koo

**Affiliations:** 1 Department of Materials Convergence and System Engineering, Changwon National University, Changwon 51140, Gyeongsangnam-do, Republic of Korea; hamidhassan774@gmail.com (H.H.); zeeshan.physics@gmail.com (Z.U.R.); 2 Dongjin Metal Co., Ltd., Changwon 51560, Gyeongsangnam-do, Republic of Korea; jdyun@djmt.kr (J.D.Y.); psw@djmt.kr (S.W.P.)

**Keywords:** fire retardant, montmorillonite clay, cotton, layer by layer (LbL) method, eco-friendly, thermal properties

## Abstract

The traditional acid-based production method was often utilized to produce flame-retardant material, which is environmentally hazardous and adversely affects the intrinsic properties of the substrates. To address these critical challenges, this work was carried out to develop a pioneer flame-retardant composite material based on cotton and biocompatible hybrid cationic/anionic species deposited through the Layer-by-Layer deposition method. The FTIR results unveil the peaks reflected by coated elements, confirm the successful deposition of coating materials on the cotton substrate. The microstructure and uniform deposition of the coating were analyzed through scanning electron microscopy. The thermal stability of the composites is enhanced with higher coating layers due to the formation of a protective char layer. Flame retardancy of the investigated samples is measured through the vertical flame test and micro-combustion calorimetry method, exhibiting remarkable reduction in peak heat release rate and total heat released rate by 47.30% and 34%, respectively. The acquired results acknowledge the suitability of the production method to produce green fire-retardant materials with excellent thermal stability and flame retardancy to utilize in the fire-resistant industry.

## 1. Introduction

Layer by layer (LbL) surface modification is a highly suitable, flexible, and relatively novel approach for depositing multilayer films on substrates of various types and geometries. Primarily introduced by Iler, who deposited alternating layers on a solid surface by following the sequential adsorption of oppositely charged colloidal particles [1]. Further, the advancement in method was achieved through the work of Decher and Hond on polyelectrolyte multilayers [2,3]. This method can be implemented through multiple mechanisms such as electrostatic interaction [4], hydrogen bonding [5,6], donor–acceptor interaction [7], covalent bonding [8], hydrophobic interactions [9], or van der Waals forces [10,11]. However, the LbL deposition involved alternately dipping the substrate into dilute water-based solutions or a combination of oppositely charged polymers or particles. Due to such a sequential dipping process, positive/negative layer pairs, referred to as bilayers (BLs), which are assembled through strong electrostatic interactions between oppositely charged molecules, seemed a promising driving force for multilayer formation, as it has minimal steric hindrance compared to others [4,12]. These interactions play a crucial role in depositing stable film assemblies.

The LbL deposition process consists of three key stages: the initial adsorption of a polyelectrolyte onto a substrate, leading to the formation of the first interfacial layer, the subsequent deposition of a second polyelectrolyte layer, resulting in the formation of a polyelectrolyte complex; and a multilayering phase, where additional layers are sequentially assembled to construct a multilayered film structure. The major force assisting the permanent attachment of the polyelectrolyte species into a multilayer micro and nanostructure can be expressed as shown below. Let us assume a solid surface carrying an electric charge density eσ per unit area [13]. The generated electric field can be expressed as follows:

(1) $$E=\left(\frac{k_{b}T}{e}\right)\left(4\pi \sigma l_{b}\right)$$

where *l_b_* represents the Bjerrum length. The electric field declines as the distance from the surface increases due to screening effects, which arise from counter-ions present in solution or additional salt ions if present [13]. This electric field creates an attractive force that pulls oppositely charged polyelectrolytes toward the solid surface, enabling the first layer to absorb. However, if the field declines due to counter-ions adjacent to the surface $\lambda =\frac{1}{2\pi \sigma l_{b}}$, this defines the region where the electric potential is most strongly influenced by the surface charge. Under these conditions, the decay of the electric field with distance z from the surface can be expressed as follows:

(2) $$E=\frac{2}{z+\lambda }$$

Equation (2) indicates a gradual, non-exponential decline governed by counter-ion distribution. However, when electrolyte salt is introduced into the solution, the screening effect is predominantly governed by these ions, causing the field to decay exponentially as follows:

(3) $$E=\frac{2}{\lambda }e^{-kz}$$

These principles govern the adsorption behavior and are critical for electrostatic LbL assembly.

The LbL has been extensively applied to deposit the coating layers on various materials, among which textiles, foams, and bulk polymers are the most widely studied [3,14]. Recently, cotton fabrics gained enormous interest of researchers to improve their fire-retardant characteristics due to their softness, breathability, and compatibility. However, to improve the fire retardancy of cotton fabrics, generally, it is treated with halogenated-based materials, which improve its fire retardancy but simultaneously pose notable dangers to both human health and environmental sustainability due to the release of harmful substances, such as hydrogen halides or formaldehyde, etc. [15]. Therefore, the development of new flame-retardant materials, which are free from harmful species such as halogen, formaldehyde, etc., is essential, as they can also meet the safety standards, minimize environmental impact, and ensure compatibility with human life [16]. To achieve these desired characteristics, eco-friendly material-based flame-retardant coatings on cotton fabrics have gained significant attention and have been proposed as suitable solutions [12], particularly those using sustainable flame-retardant compounds derived from bio-based resources, such as starch [17], phytic acid [18], gelatin [19], urea [20], lignin [21], tannic acid [22], egg white protein [23], and clay [24]. In this direction Yu-Chin Li et al. developed BPEI/MMT-coated cotton fabrics, reporting a minute enhancement in thermal stability of the produced coated cotton species [25]. Among the various biomass sources, chitosan (CH) and montmorillonite (MMT) are valuable resources and abundant in nature, as both are naturally occurring materials. Chitosan is derived from chitin, OH and NH_2_ groups, which are contained in their chemical structure, making it an eco-friendly flame retardant [26]. However, CH alone is not highly effective as a flame retardant; it can be combined with other additives to create an effective flame-retardant formulation. Additionally, MMT is characterized by negatively charged silicate sheets with nanometer-scale thickness, stacked through electrostatic interactions [27]. Due to its unique one-dimensional layered nanostructure and cation exchange properties, MMT offers significant potential for modification and a broad range of applications. In addition, MMT enhances the viscosity of coating and exhibits a synergistic effect in improving thermal stability, barrier performances and smoke suppression properties [28,29].

In this study, a flame-retardant system composed of a hybrid cationic solution, which consists of branched polyethyleneimine (BPEI) and chitosan (CH), and a water-based anionic solution of MMT was developed. When coating is applied to cotton, it imparts excellent flame retardancy and maintains the original mechanical properties of cotton fabric. In the BPEI/CH/MMT flame retarding system, cationic solution acts as dilution of flammable gases during combustion, while anionic solution facilitates the formation of a thermal barrier and contributes to the mechanical strength and stability of the char layer, to reduce the release of combustible volatile gases. Further characterization supports the enhancement of flame-retardant properties and its effective role in boosting fire safety.

## 2. Materials and Methods

### 2.1. Materials

The pristine cotton plain weave fabric substrate (600 mm × 100 mm) with mass per unit area of 180 g/m^2^ was used in this study and sourced from a local market, Changwon (Republic of Korea). Prior to treatment, the fabric underwent a washing process and was subsequently dried at 80 °C in an oven. Montmorillonite clay (MMT) (Na, Ca)_0.3_(Al, Mg)_2_Si_4_O_10_(OH)_2_·nH_2_O, surface area of 220–270 m^2^/g, chitosan (CH) (C_56_H_103_N_9_O_39_, molecular weight: 5 × 10^4^–1.9 × 10^5^ Da), and BPEI (H(NHCH_2_CH_2_)nNH_2_) average molecular weight~25,000 Mn~10,000 were procured from Sigma-Aldrich (St. Louis, MO, USA). Another reagent, including sulfuric acid (H_2_SO_4_, 95%), acetic acid, sodium hydroxide (NaOH, 92%), and deionized (DI) water, was also utilized for this study.

### 2.2. Deposition of Hybrid Cationic/Anionic Species via LbL Dipping Method

To enhance the thermal stability and fire retardancy of cotton fabrics, two solutions were formulated: an anionic and hybrid cationic solution using DI water. The anionic solution was prepared by dissolving MMT in DI water to a 2% concentration, with the pH adjusted to 10 using a 2% NaOH solution. The cationic hybrid solution was synthesized through a combined approach: by dissolving 2% CH in DI water using acetic acid and stirring at 50 °C overnight. Simultaneously, 2% BPEI was dissolved in DI water, heated to 80 °C, and then cooled to an ambient temperature and stirred overnight. A hybrid cationic solution containing both CH and BPEI was subsequently prepared for deposition, with the pH adjusted to 4.5 using a 1 M H_2_SO_4_.

**Figure 1:**
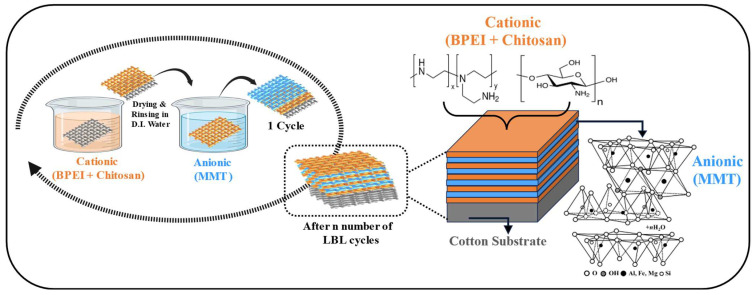
Schematic diagram of the BPEI/CH and MMT layering through the LbL method.

The LbL deposition process involves immersing cotton fabric in aqueous solutions with opposite charges, as depicted in Figure 1. Initially, the fabric samples were soaked in a cationic solution for 5 min, followed by drying at 100 °C in an oven for 10 min on a flat surface. After drying the cotton substrate is rinsed in deionized water. Then the fabric samples were immersed in an anionic solution for 5 min, followed by drying and rinsing again with DI water. This sequence of steps formed a single cycle, referred to as single bilayer. The process was repeated for 5, 10, 15, and 20 bilayers. The pH was monitored and adjusted to 4.5 and 10 after every 5 bilayers for anionic and cationic solutions, respectively. In starting bilayer immersion time in each solution was for 5 min, while remaining immersion time was 1 min. The resulting cotton fabrics were coated with C-BPCM-XBL, where C-BPCM stands for cotton-branched polyethyleneimine, chitosan, and montmorillonite clay, and X represents the number of bilayers (5, 10, 15, and 20).

### 2.3. Characterization Methods

FTIR analysis was carried out with an FT-IR-6300 spectrometer from Jasco International Co., Ltd., Tokyo, Japan. The spectra were obtained in a wavenumber range of 400–4000 cm^−1^ at a resolution of 4 cm^−1^. Each spectrum was collected from 32 scans. The thermal stability of both uncoated and coated cotton fabric samples was evaluated using a thermogravimetric analyzer (PerkinElmer Pyris-1 instrument, MA, CA, USA), under nitrogen atmosphere (20 mL/min). The analysis was performed over a temperature range of 30–700 °C with a heating rate of 20 °C/min. Each sample weighed approximately 9–12 mg. A pyrolysis combustion flow calorimeter was used in accordance with ASTM D7903 standards to assess the flame retardancy of the cotton fabrics and coatings. The furnace temperature was set at 900 °C, with an oxygen concentration of 20% (*v*/*v*) and a heating rate of 1 °C/min. The surface structures of both coated and uncoated fabric samples, as well as their char residues after the flammability test, were examined using a low-voltage scanning electron microscope (LVSEM, Merlin Compact, Helmholtz, Germany). Energy-dispersive X-ray spectroscopy (EDX) coupled with the LV-SEM was used for elemental analysis of the coating surface. To improve conductivity the samples were sputter-coated with platinum under high vacuum for about 2.5 min before the analysis. The vertical burning test was conducted on both uncoated and coated fabrics in accordance with ASTM D6413 standards in UL 94 test chamber (fire testing technology, West Sussex, UK). For testing the fabric samples with dimensions of 300 mm × 76 mm were used. The 38 mm fire source was placed 19 mm below the center of the samples and removed after 12 s.

## 3. Result and Discussion

### 3.1. FTIR Analysis

**Figure 2:**
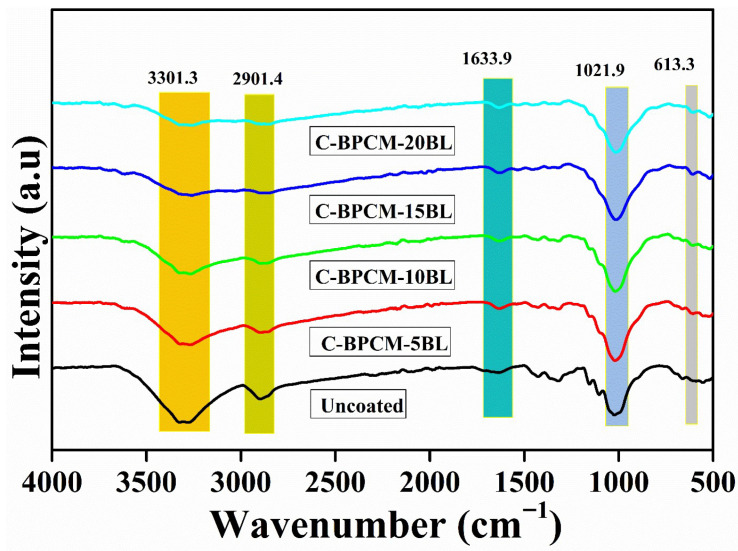
FTIR spectra of coated and uncoated cotton samples.

FTIR spectroscopy is an analytical technique employed to characterize organic, polymer-based, and certain inorganic materials by examining chemical bonds and molecular structure. It provides critical information about the molecular structure by detecting vibrations of specific chemical bonds. When a sample is exposed to infrared radiation, a distinct functional group absorbs energy at characteristic wavenumbers, resulting in unique peaks. The FTIR spectra of untreated and treated cotton fabric specimens are illustrated in Figure 2. The broad absorbance band in specimens at 3301 cm^−1^ and 2901 cm^−1^ is assigned to the stretching -OH and C-H vibration bonds, respectively [30]. However, both peaks are characteristic of cellulose, and the diminished intensity of these peaks is due to the cellulose backbone layering of cotton fibers, which suggests effective shielding of cellulose functional groups due to the deposition of coating species. Absorption band observed at 1633.9 cm^−1^ in the coated samples ascribed to the C=O vibration of the acetyl group (in the amide I region) of chitosan [31], confirming the biopolymer on the fiber surface. The band at 1021.8 cm^−1^ in the untreated cotton is attributed to the CO and OH stretching vibrations of the polysaccharide in cellulose [32]. However, in the coated samples, the peak sharpness and intensity increased, which is assigned to the Al-O-Al group because of the MMT clay [33,34]. A small new absorbance peak at 613.3 cm^−1^ is due to Si-O-Al bending vibration [35,36]. However collective spectral changes in FTIR spectra provide strong evidence that an organic–inorganic hybrid layer was effectively deposited onto the cotton fibers.

### 3.2. Surface Morphology

**Figure 3:**
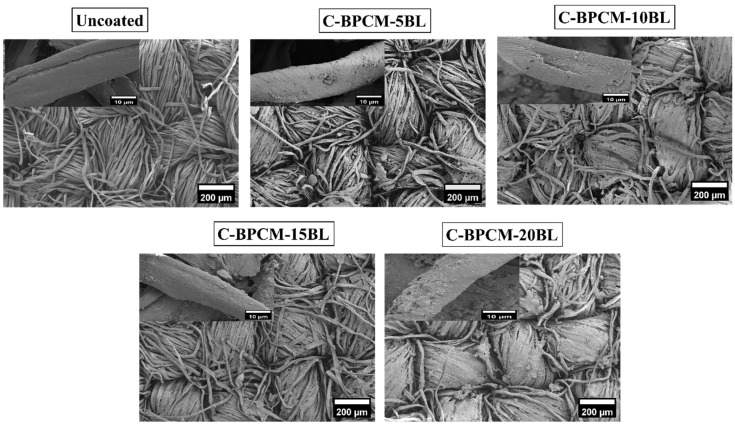
SEM analysis of uncoated and coated samples.

SEM is commonly used to examine the surface structure of solid materials in great detail. It produces detailed images of a sample by scanning it with a focused beam of electrons, which interact with the surface to provide a high-resolution morphological information [37]. Subsequently, the morphological features and surface element distributions of each sample were observed using SEM coupled with EDX and illustrated in Figure 3. As shown in Figure 3, the SEM images of the uncoated specimen show a plain-weave structure characterized by a surface that appears to be relatively smooth. However, the roughness of the surface is observed to be increased with the number of coating layers due to natural growth as reported [38]. Additionally, the gap between the fibers gradually diminished and merged as the number of deposited layers increased. Small MMT aggregates appear between the fabric fibers, possibly caused due to insufficient rinsing during the layer deposition process Figure 3, while this kind of surplus coating is often seen on the substrate with wavy surfaces, including fibers, fabrics and foams [39,40,41]. Additionally, the cotton fabric surface gradually appears more densely coated with increasing coating layers, as fewer individual fibers remain exposed within the yarn structure. However, even after 20 layers, some protruding microfibers and localized agglomerated regions are still observable in the inter yarn spaces.

**Figure 4:**
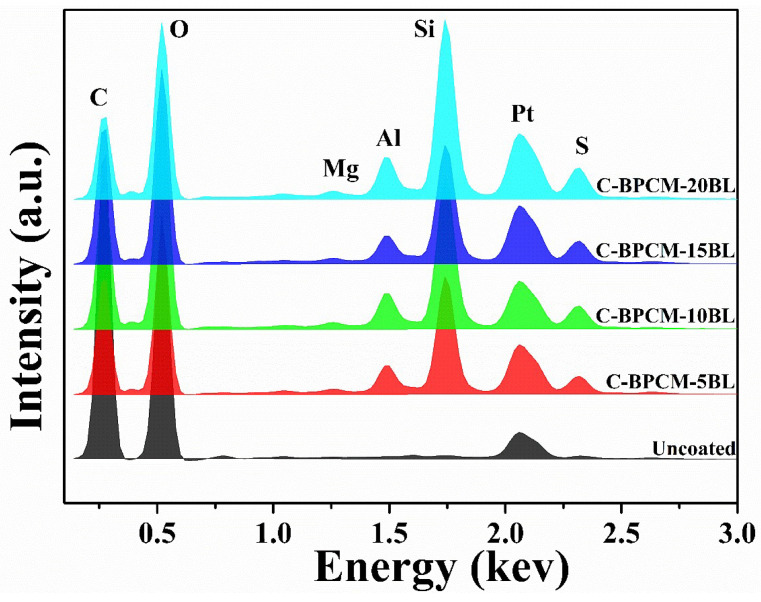
EDX analysis of coated cotton fabric samples and uncoated version.

For elemental identification and composition analysis, the EDX measurement was performed for each sample, and the resulting spectrum is shown in Figure 4 and are numerically recorded in Table 1. The EDX spectra indicated that the various existing elements, such as Al, Si, and Mg, were sourced from the clay electrolyte solution.

### 3.3. Thermal Analysis

**Figure 5:**
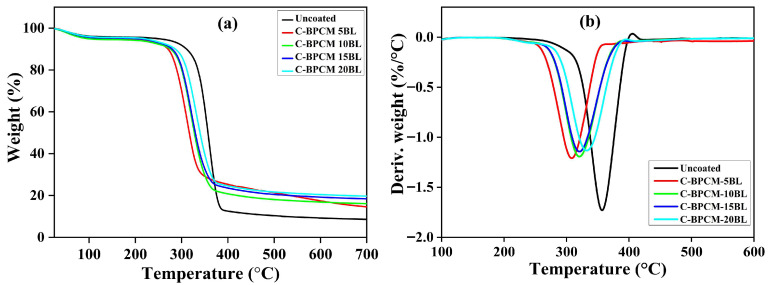
(**a**) TGA and (**b**) DTGA analysis of the samples.

The thermogravimetric analysis was performed to examine the degradation behavior of uncoated and coated samples, as illustrated in Figure 5a,b. Both untreated and treated samples exhibited a single-step degradation process. An initial weight loss below 130 °C was observed, which can be attributed to the vaporization of water and other physically attached substances on the fibers [42,43]. Subsequently, in uncoated fabric, the dominant thermal degradation event was observed in the region of 310–390 °C, with maximum weight loss recorded at 360 °C. Approximately 59.9% mass reduction was detected as shown in Figure 5b, where the high evaluation of combustible gases, abrupt ignition, and the spontaneous ignition threshold (~400 °C), which accumulated thermal energy, drive auto-combustion as reported in earlier studies [44,45,46]. Interestingly, the coated samples start early degradation, and T_onset_ temperature decreases to lower values as tabulated in Table 2. However, the coated samples exhibited slightly reduced thermal stability, as indicated by their thermal degradation curves shifting to the left compared. This early thermal degradation in flame-retarded samples facilitates the timely formation of a protective char layer, which might delay ignition [47,48]. This behavior was mainly caused by the decomposition of chitosan and branched polyethyleneimine (BPEI), which starts decomposition at approximately 247 °C and 300 °C, respectively, as reported in the literature [39,49,50]. Such premature behavior in modified samples dilutes the effect of combustible gases by release of nonflammable gases and expedites char formation, which functions as an insulating barrier and prolongs the ignition time [47]. Despite the earlier onset, the coated layers resisted mass loss at higher temperatures, resulting in a significantly higher char residue compared to the uncoated specimen. The highest char residue is found to be for the maximum 20-layer coated sample with 19.67% magnitude at 700 °C.

### 3.4. Pyrolysis-Combustion Analysis

**Figure 6:**
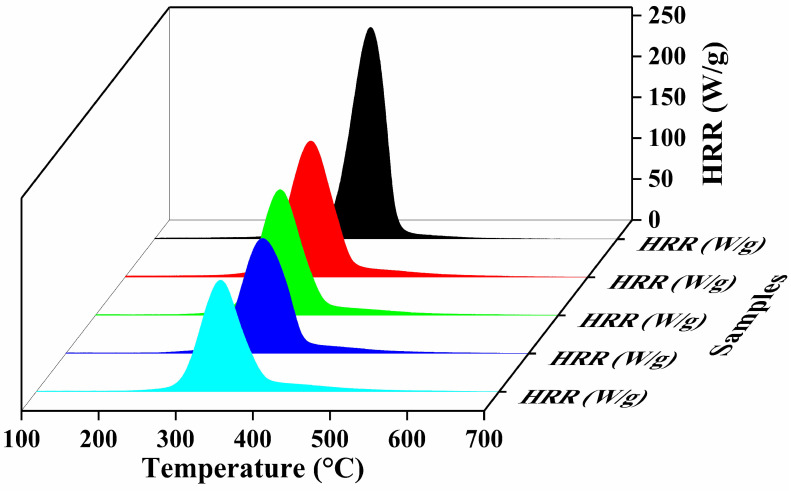
Thermal parameters measured by micro-cone calorimetry heat release rate curves (HRR).

To achieve the deep insights regarding the combustibility and fire hazardless of explored specimens, the microscale combustion calorimetry (MCC) technique was performed. The heat release rate (HRR) curves, as a function of temperature for untreated and treated cotton samples, were obtained through MCC results to evaluate the thermal combustion properties of coated and uncoated samples as shown in Figure 6. The HRR, a critical parameter in fire modeling, indicates the heat release ability of the tested sample and provides insight into its combustible behavior. A higher HRR value generally correlates with high flammability [51]. The HRR at various temperatures is influenced by factors such as the heating rate, oxygen consumption, flow rate, and the sample weight. Other key flammability parameters used to characterize fabric combustion, which include heat release capacity (HRC), peak heat release rate (pHRR) and its corresponding temperature (TpHRR), and the total heat release rate (THR), are plotted in Figure 6, and the associated values are summarized in Table 3. All the coated cotton samples have exhibited significantly lower HRR, pHRR, THR and HRC values compared to the uncoated cotton sample. The uncoated cotton fabric, being highly flammable, displayed a high pHRR value of 258.9 w/g. Compared with untreated samples, samples coated with the BPCM system demonstrated significant reduction in MCC parameters. This reduction was most pronounced in the 20BL-coated samples, which exhibit the lowest pHRR value of 136.4 (w/g). The observed decreasing trend in pHRR values with an increasing number of deposited layers suggests enhanced thermal stability, which again satisfied the TGA results. Additionally, the intensity of HRR peaks is significantly reduced and shifted toward lower temperature of the coated samples, due to pyrolysis of BPEI at a lower temperature [52]. The increased thermal stability of the coated fabrics is attributed to the heat-shielding properties of the MMT, dilution effect and char-forming ability of BPEI and chitosan. This coating effectively reduces oxygen permeation to the substrate and slows down the thermal decomposition of coated samples. Similar behavior has been previously reported for a clay nanocomposite [53,54].

### 3.5. Flammability and After-Burn Analysis

**Figure 7:**
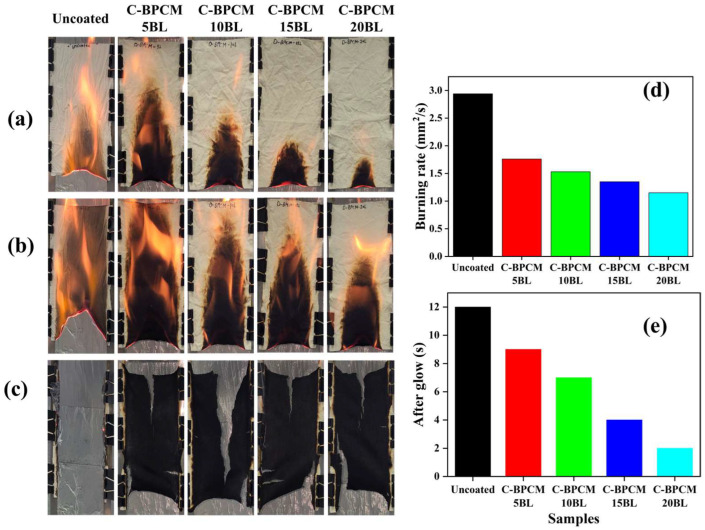
Vertical flame snapshots of the samples: (**a**) images after 5 s, (**b**) 10 s, (**c**) final images of residual char, (**d**) burning rate, and (**e**) after glow.

The flame-retardant properties of the coated fabric samples were evaluated using a vertical flame test (VFT), conducted by exposing the samples to a small flame in accordance with the ASTM D6413 standard as reported by M. Li et al. [27]. This test aimed to correlate the macro-scale thermal performance with the micro-scale data obtained from thermogravimetric analysis (TGA). The flammability of the samples is influenced by several key factors, including susceptibility to ignition, spread of flames, amount of heat released, ability to self-extinguishment, resistance to fire over time, production of smoke, release of toxic gases, etc. The VFT was performed on all samples, with optical images captured at 5 s and 10 s after the removal of the flame burner, and shown in Figure 7a,b. Final images were taken after the complete combustion of all samples, Figure 7c. The results revealed that the untreated fabric was highly flammable, igniting immediately upon contact with the flame source. Once ignited, the flame spread rapidly through the untreated fabric, resulting in complete combustion and leaving almost no carbon residue but only gray-white ash. Moreover, no flame resistance was observed in the untreated sample. On the other hand, the coated samples effectively resisted the flame propagation, substantial delay in ignition and reduced flame propagation, Figure 7d. The flame height for each sample decreased drastically. After 10 s of ignition, the 20BL exhibited the lowest flame height, and, as reported earlier, this behavior is linked to the physical barrier formed by the clay–nanoparticle matrix [47,55,56]. The coatings on the fabric samples acted as a barrier against the flame, reducing the flame spread, decreasing volatile and combustible materials, and limiting oxygen access, which prevented it from reacting with volatiles. MCC results also demonstrated a significant reduction in heat release rate (HRR) and total heat release (THR) values for the 20BL-coated sample. However, the coatings were still unable to fully withstand the flame, suggesting that complete flame retardancy cannot be predicted solely based on heat release values.

### 3.6. Surface Analysis (After Burn)

**Figure 8:**
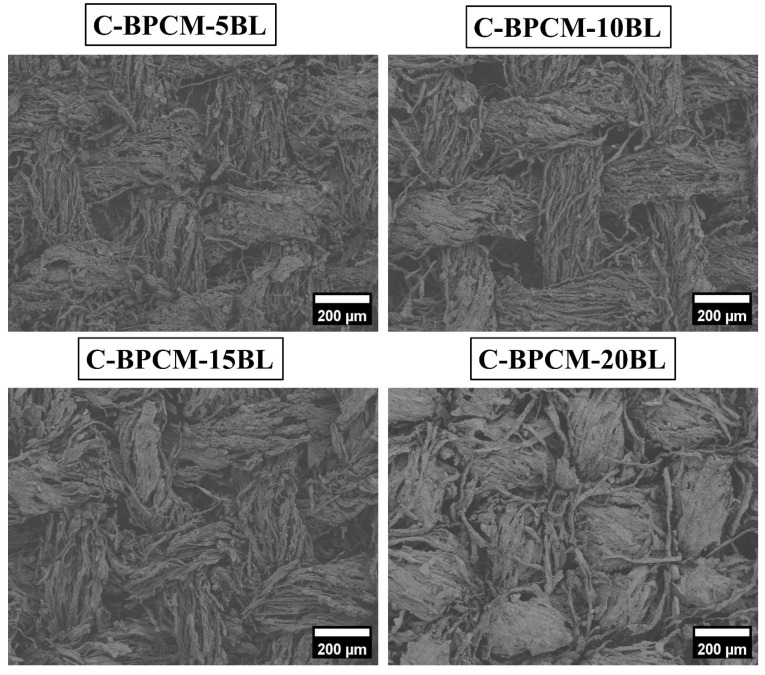
After burn surface morphologies of char residue.

The char residues of the coated cotton samples were analyzed using low-vacuum scanning electron microscopy (LV-SEM) to evaluate their structural integrity and morphology after VFT. As shown in Figure 8 (SEM images), the coated cotton samples exhibited a dense and cohesive char structure, indicating effective fire resistance. Unlike uncoated cotton, which was completely converted to ash without leaving any char residue, the coated samples retained a well-defined morphology. However, SEM images reveal tightly packed and interwoven char layers, with minimal structural deformation or fragmentation with increasing coating layers. This compact and non-porous structure suggests the successful formation of a protective barrier during combustion. Additionally, the observed char maintained its original textile framework, implying that the coatings significantly enhanced the thermal stability of the fibers under high-temperature conditions. Furthermore, coarse residue visible on the surface likely corresponds to inorganic SiO_2_ particles derived from the thermal degradation of MMT clay [57,58]. These particles likely contributed as a condensed-phase barrier, effectively enhancing fire resistance by reducing oxygen permeation and thermal degradation of the underlying polymer matrix [59].

## 4. Conclusions

The thermal stability of the uncoated cotton fabrics was significantly enhanced in this work through the deposition of the fire-retardant environment friendly materials. The cotton was covered with a distinct number of layers from 0 to 20 layers via the facile layer-by-layer dip coating method. The coated samples effectively show elevated resistance to the fire with increasing the coating layers validated through the VFT results. The highest 20-layer coated samples exhibit a 47.3% and 34% reduction in the pHRR and THR values in comparison to the uncoated cotton fabrics. This enhanced fire resistance performance is shown by the combined action of BPEI/chitosan-coated species, which releases non-flammable gases during the thermal decomposition and thereby dilutes the combustible volatiles. Additionally, the MMT clay facilitates the higher char residue formation as validated through the TGA, shows the early degradation of the coated species, initiates the fire-retardant mechanism at a lower temperature and increases the thermal stability of the cotton fabrics. Conclusively, in this work, fire retardancy of cotton fabrics was improved by using the eco-friendly materials and providing an alternative to replace the traditional hazardous halogenated materials utilized for fire-retardant applications.

## Figures and Tables

**Table 1:** Elemental composition of the cotton fabric sample after coating deposition.

Element	C-BPCM-5BL	C-BPCM-10BL	C-BPCM-15BL	C-BPCM-20BL
C	46.19	44.89	43.34	39.15
O	43.69	43.16	42.6	40.93
Mg	0.22	0.48	0.58	1.12
Al	1.59	2.63	3.41	4.26
Si	6.98	7.39	8.48	12.32
S	1.33	1.45	1.59	2.22

**Table 2:** Thermogravimetric analysis parameters of the coated and uncoated samples.

Samples	T_on set_ (°C)	T_−10%_ (°C)	T_peak_ (°C)	Residue at T_700°C_ (%)
Uncoated	270.8	310.4	357	8.6
C-BPCM-5BL	254.4	270.5	308.6	14.6
C-BPCM-10BL	239.8	275.6	320.3	16.1
C-BPCM-15BL	234.2	279.2	321	18.4
C-BPCM-20BL	224.6	286.2	332.4	19.8

**Table 3:** Combustion and pyrolysis parameters obtained from MCC analysis.

Samples	pHRR (W/g)	T_pHRR_ (°C)	THR (kJ/g)	HRC
Uncoated	258.9	379.9	15	296
C-BPCM-5BL	166.5	342.4	12.5	184
C-BPCM-10BL	153.8	340.5	11.4	172.7
C-BPCM-15BL	140.7	340.1	10.4	157.83
C-BPCM-20BL	136.4	339.2	9.9	148.75

## Data Availability

The data presented in this study are available upon request from the corresponding author due to the research data are the sole property of the university.
